# VMPANet: Vision Mamba Skin Lesion Image Segmentation Model Based on Prompt and Attention Mechanism Fusion

**DOI:** 10.3390/jimaging11120443

**Published:** 2025-12-11

**Authors:** Zinuo Peng, Shuxian Liu, Chenhao Li

**Affiliations:** 1 School of Computer Science and Technology, XinJiang University, Urumqi 830046, China; 2 Joint International Research Laboratory of Silk Road Multilingual Cognitive Computing, XinJiang University, Urumqi 830046, China

**Keywords:** skin lesion image segmentation, VMPANet, Vision Mamba, attention mechanism, prompt mechanism

## Abstract

In the realm of medical image processing, the segmentation of dermatological lesions is a pivotal technique for the early detection of skin cancer. However, existing methods for segmenting images of skin lesions often encounter limitations when dealing with intricate boundaries and diverse lesion shapes. To address these challenges, we propose VMPANet, designed to accurately localize critical targets and capture edge structures. VMPANet employs an inverted pyramid convolution to extract multi-scale features while utilizing the visual Mamba module to capture long-range dependencies among image features. Additionally, we leverage previously extracted masks as cues to facilitate efficient feature propagation. Furthermore, VMPANet integrates parallel depthwise separable convolutions to enhance feature extraction and introduces innovative mechanisms for edge enhancement, spatial attention, and channel attention to adaptively extract edge information and complex spatial relationships. Notably, VMPANet refines a novel cross-attention mechanism, which effectively facilitates the interaction between deep semantic cues and shallow texture details, thereby generating comprehensive feature representations while reducing computational load and redundancy. We conducted comparative and ablation experiments on two public skin lesion datasets (ISIC2017 and ISIC2018). The results demonstrate that VMPANet outperforms existing mainstream methods. On the ISIC2017 dataset, its mIoU and DSC metrics are 1.38% and 0.83% higher than those of VM-Unet respectively; on the ISIC2018 dataset, these metrics are 1.10% and 0.67% higher than those of EMCAD, respectively. Moreover, VMPANet boasts a parameter count of only 0.383 M and a computational load of 1.159 GFLOPs.

## 1. Introduction

Medical image diagnosis plays a crucial role in clinical practice, yet this process heavily relies on the subjective assessment of physicians, which carries a certain risk of misdiagnosis. Misdiagnosis can not only delay patient treatment but also increase mortality and morbidity rates [1]. Artificial intelligence technology has advanced continuously, allowing medical image segmentation to gradually become an essential auxiliary tool to address this issue, which holds significant importance in the medical field. This technology provides a precise image basis for computer-aided diagnosis, helping physicians more accurately identify lesions and determine the type and extent of diseases. Additionally, it can be used to evaluate post-treatment effects by comparing pre- and post-treatment images, visually presenting changes in lesion areas and providing a robust basis for subsequent treatment decisions. In summary, medical image segmentation is a crucial task that underpins computer-aided diagnosis, surgical guidance, and post-treatment evaluation.

**Figure 1:**
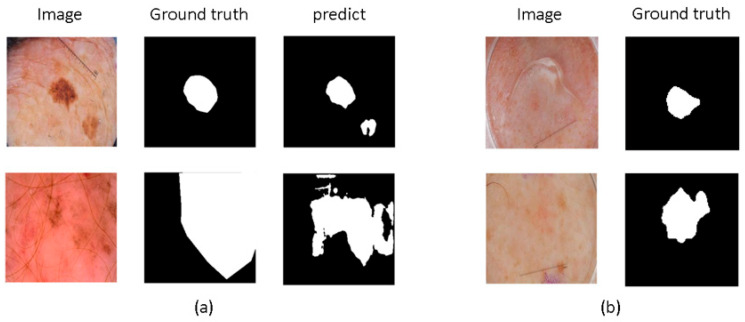
The challenges in medical image segmentation. (**a**) The limited receptive field of CNN-based methods restricts their ability to perform global modeling. (**b**) Medical images exhibit complex edge features, such as blurred boundaries and difficulty in defining boundaries.

In the field of medical image segmentation, the application of machine learning and deep learning technologies has made significant strides. Notably, methods based on Convolutional Neural Networks (CNNs), Transformers, and Mamba have shown impressive performance [2,3,4]. These technologies offer powerful tools for medical image segmentation tasks, greatly advancing the field, yet numerous challenges remain. CNN-based methods excel at capturing local texture details and enhancing feature interaction [5,6]. However, their limited receptive fields restrict their ability to perform global modeling, which may reduce accuracy, as shown in Figure 1a. To address this issue, some studies [7,8,9] have employed dilated convolutions and large kernel convolutions to enhance the model’s ability to capture long-range dependencies, attempting to overcome the limitations of CNNs’ receptive fields. Nevertheless, these methods still face limitations in fully capturing rich global context.

On the other hand, Transformers have developed rapidly, leading many researchers to combine them with medical image segmentation methods. Transformer-based methods excel in global modeling and establishing long-range dependencies. Consequently, related research [10] has applied Transformers to medical image segmentation to effectively capture global context, although they are not as effective as CNNs in extracting local details. To address this limitation, some studies [11,12,13] have combined Transformers and CNNs to perform hybrid encoding, aiming to consider both low-level features and global regions of images, thereby maintaining feature richness and consistency. However, these methods often come with high computational costs and large parameter counts. In contrast, Mamba-based architectures offer linear complexity and excel in capturing long-range dependencies [14,15]. For instance, H-vmunet [16] achieves accurate predictions by leveraging CNNs to capture detailed features at lower levels and employing high-order visual Mamba to establish long-range dependencies at deeper levels.

Moreover, as depicted in Figure 1b, medical images exhibit complex edge features. Some methods [17,18] enhance edge information by progressively integrating shallow and deep features or by stacking multiple convolutional layers to continuously extract key information. Additionally, some methods [19,20] extract high-order and low-order features from encoder outputs, capturing high-frequency edge and low-frequency non-edge information and using channel and spatial attention mechanisms to fuse them, enriching edge features. Comparatively, coarse-to-fine registration methods can also capture edge details. Meng et al. [21] designed a correlation-aware window MLP module to capture correlation-aware multi-distance dependencies with different receptive fields, utilizing correlations between images and registration steps to achieve a coarse-to-fine registration process, accurately capturing more details. However, these methods do not account for the diversity of image edge information. Zhou et al. [22] introduced multi-granularity edge detection to capture edge information at different granularities, creating multi-scale feature maps to generate various edge maps. They also decomposed feature maps into low-frequency and high-frequency components to precisely control edge map details. Although these methods are effective, they often incur high computational costs and do not specifically emphasize the details of boundary features.

We analyze the inherent complexity of different edge sizes—namely, the diverse shapes and uneven distribution of skin lesion areas and the complexity of edge structures such as blurred boundaries and difficult-to-define borders—and propose VMPANet, a lightweight network based on the combination of visual Mamba and convolution, contextual self-prompting, and attention mechanisms. Specifically, VMPANet extracts local detail feature information through convolutional layers and establishes long-range dependencies using Mamba. Additionally, it facilitates the propagation and collaboration of features at different depths based on previously generated hint masks. It also extracts multi-scale features through efficient parallel depthwise separable convolutions and adaptively aggregates each feature map at each scale using pointwise convolutions to obtain comprehensive feature representations. By leveraging edge enhancement, spatial attention, and channel attention mechanisms, VMPANet adaptively captures edge details and integrates complex spatial relationships. Furthermore, to combine cross-layer features, we employ a cross-attention mechanism, processing features from the spatial dimension, using high-level semantic cues for saliency guidance and low-level texture information for detail enhancement, dynamically allocating attention to specific regions to facilitate collaboration between cross-layer features.

Our main contributions are summarized as follows:

1. We propose a Context Prompt Encoder (PCM) that addresses the inherent complexity of different edge sizes by extracting local detail features using convolution and establishing long-range dependencies with visual Mamba. It enhances feature propagation based on previously generated hint masks, aiding in better localization of salient objects and adding fine-grained details.
2. We design a Context-Enhanced Decoder (MCA) that addresses the complexity of edge structures by capturing unified feature representations through multi-scale parallel depthwise separable convolutions. It adaptively emphasizes fine-grained edge information from spatial and channel dimensions, extracting complex spatial relationships to highlight important regions in medical images.
3. We introduce a cross-attention mechanism to accurately localize salient targets in deep features while utilizing detail cues from shallow features. The proposed Cross-Attention Fusion (CAF) module employs a single-head self-attention mechanism to effectively enhance the complementarity between semantic information in deep features and texture details in shallow features, which is crucial for accurately localizing salient objects.

To provide a clear roadmap for this paper, the subsequent sections are organized as follows. Section 2 reviews related work on CNN-based, Transformer-based, and Mamba-based models for medical image segmentation. Section 3 details the proposed VMPANet architecture, including the Context Prompt Encoder (PCM), Context-Enhanced Decoder (MCA), and Cross-Attention Fusion (CAF) module, as well as the loss function design. Section 4 presents experimental settings, datasets, evaluation metrics, and comparative results with mainstream methods, along with ablation studies to validate the effectiveness of each component. Finally, Section 5 concludes the paper and outlines future research directions.

## 2. Related Work

This section summarizes existing research relevant to medical image segmentation, focusing on three mainstream model frameworks: CNN-based, Transformer-based, and Mamba-based approaches. As illustrated in Table 1, each sub-section elaborates on the core principles, advantages, limitations, and representative works of the corresponding framework, laying a foundation for the proposed VMPANet by identifying gaps in current research.

### 2.1. Medical Image Segmentation Trends

Recent advances in medical image segmentation have witnessed the integration of emerging technologies and interdisciplinary methods, expanding the boundaries of clinical applicability and feature extraction capability. On the one hand, the fusion of cutting-edge technologies such as metaverse with healthcare has opened new avenues for multi-modal data integration and clinical workflow optimization [23]. This trend emphasizes the need for segmentation models to adapt to complex clinical scenarios, such as real-time interaction and multi-source data fusion, which aligns with our goal of enhancing VMPANet’s practical value in clinical settings. On the other hand, rule-based and decision-driven methods remain valuable complements to deep learning approaches, especially in scenarios requiring interpretable feature extraction. For instance, Gupta et al. [24] proposed a fuzzy rule-based system combined with decision trees for breast cancer detection, demonstrating the effectiveness of structured decision logic in capturing discriminative medical image features. This work highlights the importance of targeted feature modeling—an idea we extend in VMPANet through attention mechanisms and edge enhancement, tailored specifically for skin lesion segmentation.

**Table 1:** Direct comparative analysis of core frameworks for medical image segmentation.

Framework	Core Advantage	Core Limitation	Relevant Citations
CNN	Efficient extraction of local texture details; mature and stable application	Weak global context modeling; poor long-range dependency capture; insufficient edge handling	[2,5,25,26,27]
Transformer	Powerful global contextual dependency modeling; flexible capture of irregular lesion structures	High computational complexity (quadratic); excessive parameter count; inadequate local detail extraction	[10,11,13,28]
Mamba	Linear computational complexity; balanced global-local modeling capability	Insufficient adaptation to 2D visual data; weak fine-grained edge and local detail extraction	[29,30,31,32,33]

### 2.2. CNN-Based Models

Deep learning has developed rapidly, significantly enhancing the performance of models based on Convolutional Neural Networks (CNNs). Rahman et al. [25] proposed an efficient multi-scale convolutional attention decoder that enhances feature maps through unique multi-scale deep convolution blocks. This model utilizes channel, spatial, and group gated attention to capture complex spatial relationships and highlight salient regions. The cascading attention mechanism has also proven to be effective. Wu et al. [26] introduced a high-order spatial interaction model based on recurrent gated convolutions and incorporated a multi-level dimension fusion mechanism, achieving precise segmentation results and better generalization capabilities. However, the limited receptive field of convolutions restricts their ability to focus on global regions. To address this issue, Azad et al. [27] proposed a large kernel attention mechanism that expands the receptive field to capture more contextual information, effectively utilizing both high-frequency and low-frequency information to perform segmentation tasks.

### 2.3. Transformer-Based Models

Transformers have developed rapidly, leading many researchers to combine them with medical image segmentation methods. Valanarasu et al. [28] used Transformers for medical image segmentation tasks, introducing a gated axial attention mechanism to effectively integrate global contextual information, particularly excelling in handling complex anatomical structures and multimodal data. Cao et al. [10] leveraged the hierarchical architecture and sliding window technique of the Swin Transformer to design a pure Transformer model for medical image segmentation, demonstrating strong capabilities in handling images of different resolutions. Compared to pure Transformer methods, combining Transformers with CNNs can achieve higher performance. Chen et al. [11] combined the powerful encoding capabilities of the Transformer structure with the classic U-Net decoder. They introduced the global feature extraction capability of Transformers, effectively capturing long-range dependencies in complex medical image segmentation tasks and significantly improving segmentation accuracy and robustness. Heidari et al. [13] further expanded the application of Transformers in medical image segmentation by constructing hierarchical multi-scale feature representations, capturing both local details and global contextual information of images, and establishing effective feature fusion between coarse-grained and fine-grained feature representations, achieving excellent performance.

### 2.4. Mamba-Based Models

**Figure 2:**
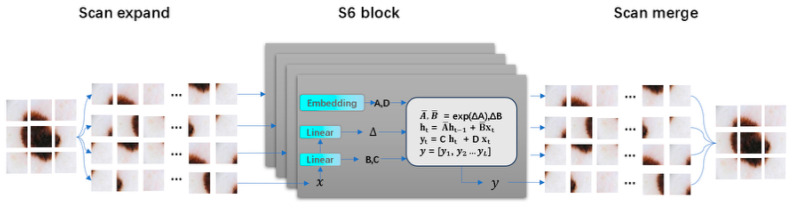
The scan expanding operation and scan merging operation in SS2D [30].

State Space Models (SSMs) have developed rapidly, prompting many researchers to combine them with medical image segmentation. Zhu et al. [29] broke through the bottleneck of traditional visual modeling with a bidirectional state space model, achieving linear complexity in global feature extraction. Its unique bidirectional recurrent structure can simultaneously capture forward and backward contextual dependencies of images, significantly enhancing model efficiency and performance. Liu et al. [30] further introduced a cross-scan mechanism and a two-dimensional selective scan module (SS2D) based on visual Mamba. As shown in Figure 2, SS2D achieves multi-scale feature fusion through four-directional scan paths, further improving model performance. Ruan et al. [31] were the first to combine a pure state space model with the classic U-Net structure, designing the Visual State Space (VSS) block as a core component. Its asymmetric encoder and decoder structure reduces the number of convolutional layers while utilizing the global modeling capability of the state space model to capture long-range dependencies. Liu et al. [32] further explored the value of transfer learning in medical image segmentation. This model uses Vmamba [30] as the encoder foundation, combined with a hierarchical feature extraction strategy, and initializes network parameters through ImageNet pretraining, significantly enhancing the model’s generalization ability. Zou et al. [33] proposed a hybrid architecture based on Mamba and CNN, achieving accurate segmentation results on a skin lesion segmentation dataset.

## 3. Method

This section comprehensively describes the design of VMPANet, a lightweight network tailored for skin lesion image segmentation. We first introduce the overall architecture of the model, then detail the three core modules (PCM, MCA, CAF), respectively, including their structural designs, working mechanisms, and mathematical formulations. Finally, the loss function adopted in the training process is presented to address the specific challenges of skin lesion segmentation.

**Figure 3:**
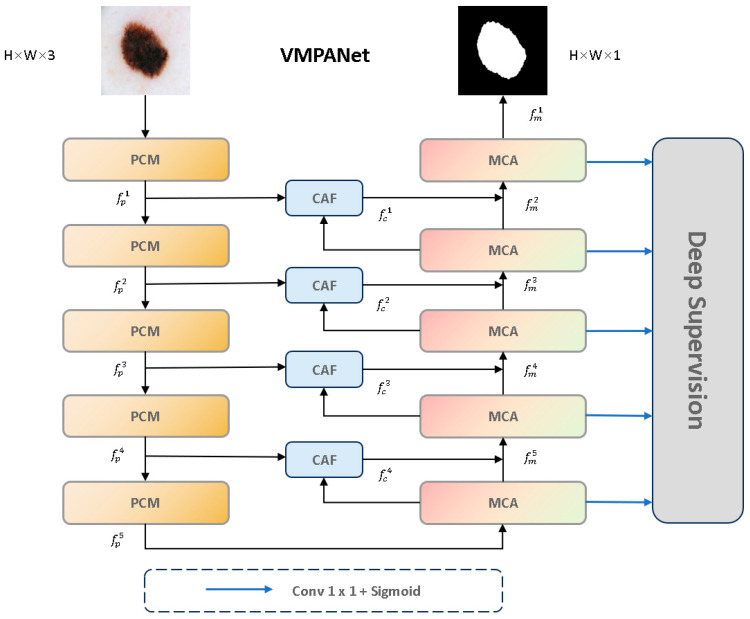
The overall architecture of VMPANet.

To address the issues of varied sizes and shapes of skin lesion areas, uneven distribution, and the difficulty in defining blurred boundaries, we propose VMPANet, a lightweight network that combines visual Mamba and convolution, contextual self-prompting, and attention mechanisms. As illustrated in Figure 3, VMPANet consists of three main modules: the PCM module, the MCA module, and the CAF module.

The PCM module is responsible for extracting local features using convolutional layers and performing global modeling with visual Mamba to obtain high-quality encoded features. Subsequently, two sets of hint masks are used to enhance feature propagation and provide target information. The MCA module first uses visual Mamba and the two-dimensional selective scan block (SS2D) to model long-term dependencies of each feature in four directions. Then, convolution operations are employed to aggregate information from the remaining four diagonal directions to enhance feature representation. Additionally, to effectively capture and aggregate multi-scale feature representations, a set of parallel depthwise separable convolutions with different kernel sizes is used to achieve this goal, effectively extracting multi-scale features. It dynamically allocates local attention to specific regions from both spatial and channel dimensions, enabling the capture of edge structures. The CAF module employs a single-head self-attention mechanism to capture global context across spatial dimensions. It uses deep high-level semantic cues from the MCA module as saliency guidance while supplementing shallow low-level texture details generated by the PCM module, thereby thoroughly highlighting globally interesting regions. Furthermore, we introduce a multi-stage supervision mechanism to guide the decoder output at each stage.

### 3.1. Context Prompt Encoder (PCM)

The challenges in skin lesion image segmentation primarily arise from the inherent complexity of varying edge sizes. Therefore, we need a mechanism that can extract both global and local contextual information while promoting effective feature propagation to fully utilize the features extracted by the encoder.

**Figure 4:**
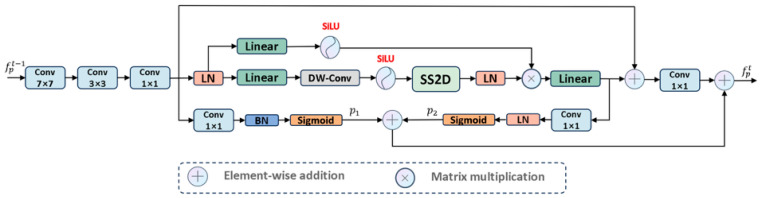
The architecture of PCM module.

As shown in Figure 4, the PCM module adopts an inverted pyramid convolution approach to extract both global and local contextual information, effectively transitioning from a large receptive field to a small receptive field. Since the receptive field of convolutional layers is limited and can only handle intra-domain information, failing to capture extra-domain information effectively, we utilized visual Mamba to extract long-range dependencies. To propagate the features extracted by the PCM module effectively, we designed prompts based on previous studies [34,35,36]. Specifically, these prompts generate a hint $p_{1}\in R^{1\times H_{i}\times W_{i}}$ based on the local detail information extracted by CNN to enhance the model’s localization ability and sensitivity to details. Simultaneously, another hint $p_{2}\in R^{1\times H_{i}\times W_{i}}$ is generated based on the global contextual information extracted by visual Mamba to guide the model in locating globally salient targets. Additionally, as shown in Figure 4, the input image size is 3 × 256 × 256, and the encoder network consists of 5 layers. The output of each encoder block is defined as $f_{p}^{i}\in R^{C_{i}\times H_{i}\times W_{i}}$, where $C_{i=\{1,2,3,4,5\}}=8\times i$, $H_{i}=\frac{256}{2^{i-1}}$ and $W_{i}=\frac{256}{2^{i-1}}$.

PCM: Extracts information from the feature space in a coarse-to-fine manner. First, we used large kernel convolutions to extract rich global detail prior information and project the input feature map $x\in R^{C\times H\times W}$ to $\hat{x}\in R^{C\times \frac{H}{2}\times \frac{W}{2}}$. Then, we used small kernel convolutions to capture local salient regions from $\hat{x}$. Finally, 1×1 convolutions are used to aggregate features from each channel, resulting in a unified feature representation $f_{ipc}\in R^{C\times \frac{H}{2}\times \frac{W}{2}}$.

Considering the limitations of convolution operations in exploring features within a limited domain, we introduced visual Mamba to capture global long-range dependencies, allowing the extraction of comprehensive feature representations $f_{m}\in R^{2C\times \frac{H}{2}\times \frac{W}{2}}$. Subsequently, we applied 1×1 convolutions to enhance feature expression capability and map $f_{m}$ to $f_{e}^{t}\in R^{2C\times \frac{H}{2}\times \frac{W}{2}}$, as shown in Equations (1) and (2):

(1) $$F\left(v_{1},v_{2},x\right)=v_{1}\times VisionMamba\left(x\right)+v_{2}\times x$$

(2) $$f_{e}^{t}=Conv_{1\times 1}\left(F\left(\lambda_{1},\lambda_{2},f_{ipc}\right)\right),\;\lambda_{i}\in (0,1]$$

where $\lambda_{1}$ and $\lambda_{2}$ are hyperparameters used to manage the influence of global and local contexts on the results, effectively balancing the two types of information.

Most importantly, to propagate features effectively, we designed self-prompts. Specifically, as shown in Figure 4, based on local features $f_{ipc}$, we established a “many-to-one channel” mapping to generate mask prompt $p_{1}$ to provide target detail information. Similarly, based on global features $f_{m}$, we also established a mapping to generate mask prompt $p_{2}$ to provide salient region information. To enhance the adaptability and stability of these two mask prompts, we normalized them to obtain standardized mask prompts. These two segmentation masks propagate from shallow to deep layers, and then synergize with $f_{e}^{t}$, effectively handling contextual features to achieve precise localization of salient targets. The specific process is as follows:

(3) $$p_{1}=Sigmoid(BN(\sum_{i=1}^{C}\omega_{i}⊙f_{ipc}^{i})),\;\;p_{2}=Sigmoid(LN(\sum_{i=1}^{C}\omega_{i}⊙f_{m}^{i}))$$

(4) $$f_{p}^{t}=f_{e}^{t}+\sum_{i=1}^{2}\left(\lambda_{i}*p_{i}\right)$$

where C denotes the number of channels, $\omega_{i}$ is used to control the importance of the feature map in the i-th channel, $f_{m}^{i}$ represents the feature map of $f_{m}$ in the i-th channel, and $f_{ipc}^{i}$ represents the feature map of $f_{ipc}$ in the i-th channel. $BN\left(\cdot \right)$ denotes batch normalization, $LN\left(\cdot \right)$ denotes layer normalization, and $\lambda_{i}$ is a hyperparameter. $f_{p}^{t}$ is used as the input for the CAF module and the next PCM module.

### 3.2. Context Enhanced Decoder (MCA)

The challenges in skin lesion image segmentation primarily stem from the complexity of edge structures. To address this issue, Ref. [8] introduced a parallel axial attention mechanism to explore multi-scale information through different convolutional depths and used an uncertainty-enhanced contextual attention mechanism to extract critical information from these features. Another study [25] utilized skip connections to fully integrate high-level salient information with low-level local information, achieving more accurate feature representation. Although these studies considered both global and local contexts, they overlooked the importance of fine-grained edge features in salient regions.

**Figure 5:**
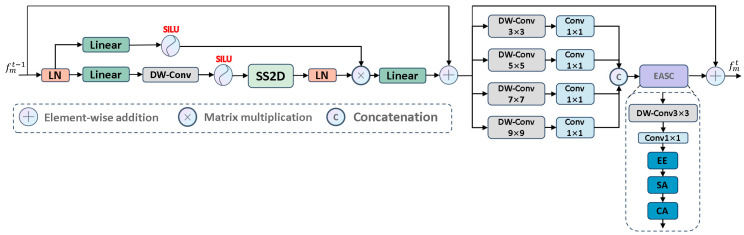
The architecture of MCA module.

Therefore, there is an urgent need for a method that can extract fine-grained multi-scale contextual information while learning complex tissue edge information, reweighting salient and non-salient information, and maintaining a lightweight model. Based on these considerations, we designed the MCA module, as shown in Figure 5. To effectively capture multi-scale feature representations, the MCA module first uses visual Mamba to establish long-term dependencies. The 2D Selective Scanning Block (SS2D Block) models long-term dependencies for each feature in four directions. To capture multi-scale detail information in hierarchical features and effectively address the direction sensitivity issue in 2D visual data, we used parallel depthwise separable convolutions to extract multi-scale features with different receptive fields and fine granularity, and aggregate information from the remaining four diagonal directions to enhance feature representation. Within each scale, it applies pointwise convolution to adaptively aggregate each feature map at that scale, achieving comprehensive feature representation. This approach aims to capture local texture details while better aggregating long-term dependencies. Next, we used the EASC module to adaptively extract important information from multi-scale features to enhance segmentation performance. Specifically, a combination of Edge Enhancement (EE), Spatial Attention (SA), and Channel Attention (CA) was used to improve the contrast between salient and non-salient regions from spatial and channel dimensions.

MCA: As shown in Figure 5, it uses four different receptive fields to map features to four different scales $X_{i}\in R^{\frac{C}{4}\times H\times W}\left(i=1,2,3,4\right)$, thereby capturing both global context and local fine-grained information simultaneously. To effectively integrate features from different channels, we introduced a 1×1 convolution to enhance feature representation, defined as follows:

(5) $$D\left(k,v\right)=Conv_{1\times 1}\left(DWConv_{k\times k}\left(v\right)\right)$$

(6) $$\begin{array}{r} f_{vm}^{t}={VisionMamba(f}_{p}^{t}) \end{array}$$

(7) $$X_{i}=D\left(2i+1,f_{vm}^{t}\right),\;i=1,2,3,4$$

Then, we used a concatenation operation to aggregate the multi-scale features $X_{i}$ into a unified feature representation, which can contain long-range dependencies and local texture details.

(8) $$\begin{array}{r} f_{cf}=Concat\left(\left[\;X_{1};\;\ldots \;;\;X_{4}\;\right]\right) \end{array}$$

where $f_{cf}\in R^{C\times H\times W}$ serves as the input to the EASC module. Considering the impact of complex boundary structures, we improved segmentation accuracy by deeply mining the multi-scale features $f_{cf}$. Specifically, we used 3×3 convolutions to extract embedded contextual information from $f_{cf}$, thereby expanding the interaction between features. This process generates rich contextual clues and salient information, as follows:

(9) $$\begin{array}{rr} & \hat{f_{cf}}=BN\left(Conv_{1\times 1}\left(DWConv_{3\times 3}\left(f_{cf}\right)\right)\right) \end{array}$$

where $BN\left(\cdot \right)$ denotes batch normalization, and $\hat{f_{cf}}$ serves as the subsequent input.

**Figure 6:**
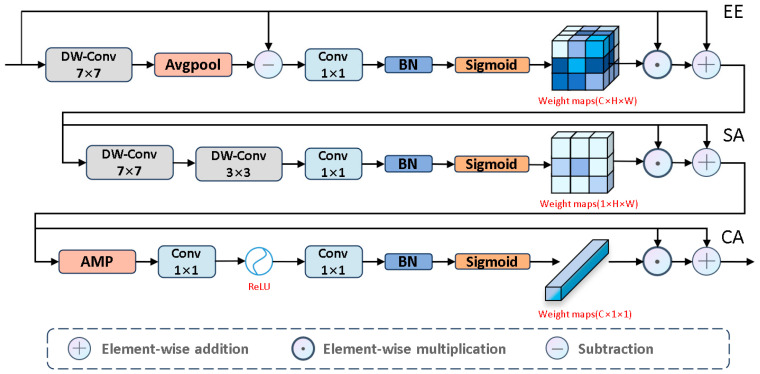
The architecture of EASC module.

Next, to emphasize salient edges, we propose the EE module, as shown in Figure 6. We used large kernel depthwise separable convolutions to capture global detail information. Then, we analyzed the edge differences between salient and non-salient information to highlight edge features. To prevent excessive edge differences, we normalized these differences to make them smooth. Next, we applied the Sigmoid activation function to estimate edge saliency scores, where higher weights correspond to salient regions and lower weights correspond to non-salient regions. Based on these scores, we further learn the boundary information of salient regions. The specific process is as follows:

(10) $$\bar{x}=BN\left(x-AP\left(DWConv_{7\times 7}\left(x\right)\right)\right)$$

(11) $$f_{ee}=Sigmoid\left(Conv_{1\times 1}\left(\bar{x}\right)\right)⊙x+x$$

where x is $\hat{f_{cf}}$, −  denotes element-wise subtraction, AP(·) denotes 3×3 average pooling, and $f_{ee}\in R^{C\times H\times W}$ serves as the input to the SA module.

To effectively capture complex spatial relationships, we introduce the SA module, which focuses on the salient regions of the input image, as shown in Figure 6. Specific steps include: first, we use large kernel depthwise separable convolutions to extract key global background information, thereby enhancing the model’s ability to accurately identify salient targets in complex environments. Next, we use small kernel convolutions to focus on local salient features. Finally, through the Sigmoid activation function, these features are converted into spatial saliency scores, assigning higher scores to salient regions and lower scores to background information. The specific process is as follows:

(12) $$s_{t}=BN\left(DWConv_{k\times k}\left(s_{t-1}\right)\right),\;t=1,2,3$$

(13) $$f_{sa}=Sigmoid\left(\eta \left(Conv_{1\times 1}\left(s_{2}\right)\right)\right)⊙\;x+x$$

where $s_{0}$ is $f_{ee}$, $\left(t,k\right)=\{\left(1,7\right),\left(2,3\right),\left(3,1\right)\}$, and $f_{sa}\in R^{C\times H\times W}$ serves as the input to the CA module.

Next, we used the CA module, as shown in Figure 6, to assign different levels of importance to each channel, thereby enhancing feature diversity to emphasize valuable features and suppress less important ones. The entire process is as follows:

(14) $$w=ReLU\left(Conv_{1\times 1}\left(AMP\left(x\right)\right)\right)$$

(15) $$f_{easc}=Sigmoid\left(Conv_{1\times 1}\left(w\right)\right)⊙\;x+x$$

where x is $f_{ca}$, AMP denotes adaptive max pooling, and $f_{easc}\in R^{C\times H\times W}$ is the output of the EASC module. To help the information flow at different depths locate salient regions in complex tissues, we established skip connections to seamlessly integrate $f_{easc}$ into $f_{vm}^{t}$. The specific process is as follows:

(16) $$\begin{array}{r} f_{m}^{t}=f_{easc}+f_{vm}^{t} \end{array}$$

where $f_{m}^{t}\in R^{C\times H\times W}(t\in \{1,2,3,4,5\})$ is the output of the *t*-layer MCA module and serves as the input to the CAF module.

### 3.3. Cross Attention Fusion Module (CAF)

To accurately locate salient targets in deep features while leveraging detailed cues from shallow features and maintaining the lightweight nature of the model, we need to develop a method that can effectively utilize cross-layer features. Thus, we propose the CAF module. This module employs a single-head self-attention mechanism to capture global context across spatial dimensions, using deep high-level semantic cues from the decoder module as saliency guidance while supplementing shallow low-level texture details generated by the encoder module. This thoroughly highlights globally interesting regions. The CAF module establishes a bidirectional information transmission channel between high-level semantic features (saliency) and low-level detail features (details). Through an attention weight matrix, it dynamically adjusts the spatial and channel dependencies between features, allowing high-level features to supplement detail information and low-level features to receive semantic guidance. The final output is a fused feature that combines spatial details and semantic discriminative power.

**Figure 7:**
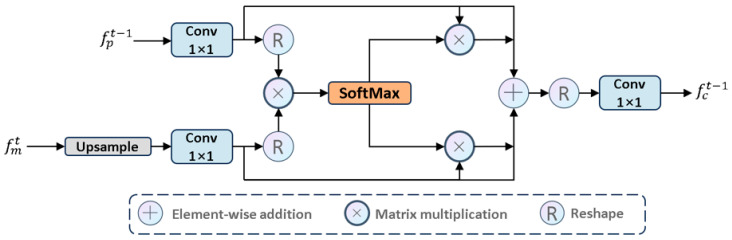
The architecture of CAF module.

CAF: Specifically, we propose the CAF module to process $f_{p}^{t}$ and $f_{m}^{t+1}$, as shown in Figure 7. We first performed an upsampling operation and then executed a 1×1 convolution to map $f_{m}^{t+1}\in R^{2C\times \frac{H}{2}\times \frac{W}{2}}$ to Q, while enhancing the CAF module’s ability to learn from $f_{m}^{t+1}$. The specific steps are as follows:

(17) $$\begin{array}{rr} & Q=BN\left(Conv_{1\times 1}\left(Up\left(f_{m}^{t+1}\right)\right)\right) \end{array}$$

where $Q\in R^{C\times N}$ is the query. Simultaneously, we also used a 1×1 convolution to adaptively learn $f_{p}^{t}$ to enhance its expressive ability and similarly map it to K. Specifically:

(18) $$\begin{array}{rr} & K=BN\left(Conv_{1\times 1}\left(f_{p}^{t}\right)\right) \end{array}$$

where $K\in R^{C\times N}$ is the key (index). Then, to capture important information across the entire spatial dimension, we used Q as $V_{1}$ and $f_{p}^{t}$ is $V_{2}$, which allows better handling of different features. Specifically, we performed matrix multiplication on Q and K, then applied the SoftMax function to obtain a set of weights that focus on salient regions and suppress non-salient regions. To enhance stability during the training process, we introduced a normalization factor τ to normalize the attention weights. Specifically:

(19) $$\begin{array}{r} A\left(Q,K,V\right)=SoftMax\left(\frac{Q⊗K^{T}}{\tau }\right)⊗V \end{array}$$

where $\tau =\sqrt{d\_k}$, and ⊗ denotes matrix multiplication. To globally extract deep high-level semantic information and capture shallow texture details, thereby promoting cross-layer feature complementarity, we designed two branches: $\left(Q,K,V_{1}\right)$ and $\left(Q,K,V_{2}\right)$. This ensures that the complementary knowledge generated by Q and K can be fully propagated across the branches, establishing connections between the complementary knowledge, $V_{1}$ and $V_{2}$, and focusing on salient regions. Specifically:

(20) $$\begin{array}{r} f_{a}=A\left(Q,K,V_{1}\right)+A\left(Q,K,V_{2}\right) \end{array}$$

Considering the limitations of the single-head self-attention mechanism in 2D modeling, we integrated feature maps using 1 × 1 convolution from a local modeling perspective, balancing high-frequency salient information and low-frequency non-salient information in each feature map. Specifically:

(21) $$\begin{array}{r} f_{c}^{t}=\eta \left(Conv_{1\times 1}\left(f_{a}\right)\right) \end{array}$$

where $f_{c}^{t}\in R^{C\times H\times W}\left(t\in \{1,2,3,4\}\right)$ is the output of the CAF module and serves as the input to the t-layer MCA module.

### 3.4. Loss Function

During the training phase, we attached pixel-level supervision to each decoder block (i.e., deep supervision strategy). By supervising at different scales, the model can capture abstract features at various levels, thereby improving accuracy and achieving fast convergence. Specifically, we supervise $f_{s}^{t}$ by combining the Binary Cross-Entropy (BCE) loss with the Dice loss, as follows:

(22) $$\begin{array}{r} L_{BCE}=\lambda_{1}\left(-\frac{1}{N}\sum_{1}^{N}\left[y_{t}log\left(f_{s}^{t}\right)+\left(1-y_{t}\right)log\left(1-f_{s}^{t}\right)\right]\right) \end{array}$$

(23) $$\begin{array}{r} L_{Dice}=\lambda_{2}\left(1-\frac{2\left|y_{t}\cap f_{s}^{t}\right|}{\left|y_{t}\right|+\left|f_{s}^{t}\right|}\right) \end{array}$$

(24) $$\begin{array}{r} L_{total}=L_{BCE}+L_{Dice} \end{array}$$

where N is the total number of samples, $y_{t}$ represents the ground truth, and $f_{s}^{t}$ is the predicted value. The parameters $\lambda_{1}$ and $\lambda_{2}$ are hyperparameters used to adjust the weights between $L_{BCE}$ and $L_{Dice}$.

## 4. Experiments and Results

This section validates the performance of VMPANet through systematic experiments. We first describe the datasets used for evaluation, followed by details of the experimental environment and parameter settings. Then, we present the evaluation metrics employed to assess segmentation quality. Comparative experiments with nine mainstream methods and ablation studies were conducted to verify the superiority and effectiveness of the proposed model, with results analyzed both quantitatively and qualitatively.

### 4.1. Datasets

We evaluated our proposed method on two datasets: ISIC 2017 and ISIC 2018. The ISIC17 dataset [37] and ISIC18 dataset [38] are publicly available skin lesion segmentation datasets from the International Skin Imaging Collaboration 2017 and 2018 challenges, respectively, containing 2150 and 2694 dermoscopic images with segmentation mask labels. Following previous work [39], as illustrated in Table 2, we split the datasets into training and testing sets in a 7:3 ratio. Specifically, for the ISIC17 dataset, the training set consisted of 1500 images, and the test set consisted of 650 images. For the ISIC18 dataset, the training set contained 1886 images, while the test set contained 808 images.

For the PH2 dataset [40], we acquired 200 images as well as dermatoscope images with segmentation mask labels. All 200 images were used for external validation. The initial size of the images was 768 × 560 pixels, and we standardized the size to 256 × 256 pixels for inputting into the model.

### 4.2. Experimental Environment

The experiments were implemented using Python 3.8 and Pytorch 1.13.0. All experiments were conducted on a single NVIDIA RTX 3090 (NVIDIA; Santa Clara, CA, USA) with 24 GB of VRAM. To ensure a fair comparison of model performance, the same data augmentation operations were applied, including horizontal and vertical flips, as well as random rotations. The hyperparameter optimizer adopted was AdamW, with the following specific settings: learning rate (lr) = 0.001, betas = (0.9, 0.999), epsilon (eps) = 1 × 10^−8^, weight decay = 1 × 10^−2^, and AMSGrad = False. For learning rate scheduling, we used the CosineAn-nealingLR strategy, where the maximum number of iterations (T_max) = 50, the minimum learning rate (eta_min) = 0.00001, and the index of the last epoch (last_epoch) = −1. Each input image was resized to 256 × 256. The training was conducted for 200 epochs, The batch size was set to 8.

Additional Explanation: Definition and Values of Hyperparameters in the Formulas: For the undefined parameters λ and ω in Formulas (1)–(4) and (22)–(24), their values are as follows: Formula (1)–(2) (Global-Local Feature Fusion): Parameters: λ_1_ (Weight of global Vision Mamba features), λ_2_ (Weight of local convolutional features); Values: λ_1_ = 0.5, λ_2_ = 0.5.

Formula (3) (Mask Hint Generation): Parameters: ω_i_ (Weight of the i-th feature channel); Explanation: These are not manually set hyperparameters, but rather learnable convolutional kernel weights of the 1 × 1 convolutional layers prompt1/prompt2 in the class. End-to-end optimization is performed during training to adaptively emphasize edge/saliency-related channels. Formula (4) (Hint-Feature Fusion): Parameters: λ_1_ (weight of p_1_), λ_2_ (weight of p_2_); Values: λ_1_ = λ_2_ = 1. Formulas (22)–(24) (Loss Functions): Parameters: λ_1_ (weight of BCE loss), λ_2_ (weight of Dice loss); Values: λ_1_ = λ_2_ = 1.

### 4.3. Evaluation Metrics

For medical image segmentation, we adopted five widely used evaluation metrics to assess segmentation performance: mean Intersection over Union (mIoU), Dice Similarity Coefficient (DSC), Accuracy (Acc), Specificity (Spe), Sensitivity (Sen), and HD95.

(25) $$mIoU=\frac{TP}{TP+FP+FN}$$

(26) $$DSC=\frac{2\times TP}{2\times TP+FP+FN}$$

(27) $$Acc=\frac{TP+TN}{TP+TN+FP+FN}$$

(28) $$Spe=\frac{TN}{TN+FP}$$

(29) $$Sen=\frac{TP}{TP+FN}$$

(30) $$HD95=MAX\{\;d_{\mathrm{xy}},\;d_{\mathrm{yx}}\;\}$$

where TP denotes True Positives, FP denotes False Positives, TN denotes True Negatives, and FN denotes False Negatives.

### 4.4. Results and Analysis

First, to evaluate the impact of different layer weights on deep supervision, we conducted experiments only on a subset of the training set’s validation set. The complete training set was divided into two parts: a sub-training set (80%) used to train the model with different weight combinations (ISIC2017: 1200 images; ISIC2018: 1509 images) and a sub-validation set (20%) used solely for performance evaluation (ISIC2017: 300 images; ISIC2018: 377 images); the test set was not involved in this process.

As shown in Table 3, the model achieves optimal accuracy only when the weights of shallow and deep layers are equal. This indicates that shallow and deep features are equally important during model training. Therefore, we adopted a third parameter setting for training.

Table 4, Table 5 and Table 6 present a comprehensive comparison of performance on the ISIC2017, ISIC2018, and PH2 datasets, while Table 7 compares model size and computational cost.

On the ISIC17 dataset, our method achieved the highest values on key metrics. Notably, its Sen value was 1.73 percentage points higher than the closest model, demonstrating superior lesion region identification ability. Similarly, on the ISIC18 dataset, our method also performed well on mIoU, DSC, Acc, and Spe, further validating its generalization ability. Although slightly inferior to some models in Sen value, its overall balance and robustness remain excellent. To verify cross-dataset generalization, we conducted zero-shot transfer experiments: the model was trained solely on ISIC2017 without any fine-tuning, domain adaptation, or parameter updates, and directly deployed for segmentation on PH2 (an independent dataset unrelated to ISIC). As shown in Table 8, our model outperformed other mainstream models on multiple metrics, demonstrating superior generalization ability. Notably, although Ultralight vm-unet [42] has fewer parameters (0.049 M), its mIoU on ISIC2017 (77.59%) and PH2 (84.23%) was 3.13% and 1.37% lower than VMPANet, demonstrating that our model achieves a better balance between efficiency and performance.

Moreover, as shown in Table 7 and Table 8, our proposed method showcased significant advantages in computational resources and model size: it requires only 1.159 GFLOPS of computational power and 0.383 M parameters, yet delivers high-precision performance. Compared to existing mainstream methods, our approach substantially reduces computational overhead and parameter count while sustaining competitive accuracy levels. These results indicate that our model is well-suited for resource-constrained environments and real-time processing scenarios.

**Figure 8:**
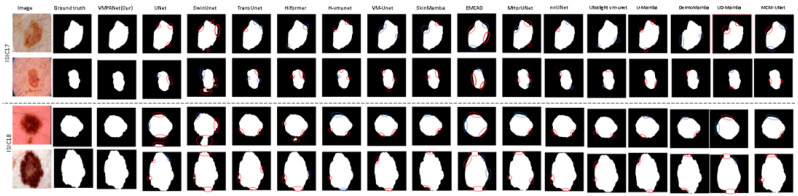
Visual comparison of sixteen methods on ISIC17 and ISIC18 datasets. (In the diagram, red circles indicate oversegmentation, and blue circles indicate undersegmentation).

We also conducted a visual comparison of our method against sixteen mainstream approaches on the ISIC17 and ISIC18 datasets, as shown in Figure 8. Most methods struggle with segmenting the complex edge structures of lesions, whereas VMPANet demonstrates superior robustness in handling these complexities. Many methods face challenges with low contrast and background interference, but VMPANet effectively addresses these issues, producing smoother and more accurate contours and boundaries. Overall, our method emphasizes both global and local information, as well as multi-scale information at different granularities, to capture significant targets in medical images. While edge enhancement can learn the differences between edge and non-edge regions to complement target contour details, it introduces errors, making it difficult for models to capture fine details in low-contrast and complex edge scenarios. Our spatial and channel attention mechanisms enable the model to adaptively focus on important targets, enhancing robustness against interference. Furthermore, we incorporate a self-attention mechanism to facilitate the complementarity between deep semantic information and shallow detail information, thereby thoroughly identifying significant targets in complex tissues.

### 4.5. Ablation Study

To comprehensively evaluate the effectiveness of our proposed method, we conducted ablation studies on VMPANet using the ISIC17 and ISIC18 datasets, as detailed below.

Table 9 shows the contributions of PCM, MCA, and CAF, while Table 10 further illustrates the ablation effects of more refined components. Clearly, as different component modules are gradually integrated into the network, segmentation performance is significantly improved, fully validating the effectiveness of each component in the model. Specifically, in PCM, convolution and Mamba operations assist the model in extracting rich local and global features, while the Prompt mechanism promotes feature propagation to identify salient targets. In MCA, depthwise separable convolutions extract multi-scale features from different receptive fields, providing rich feature representations for subsequent processing. The EASC module effectively fuses multi-scale features, while the EE module extracts complex edge features to locate salient targets. The SA and CA modules focus on salient regions, extracting key features. The CAF module extracts common salient regions from cross-layer features, promoting complementarity between deep semantic information of salient targets and detailed semantic information of shallow salient targets.

## 5. Conclusions

In this paper, we propose an effective medical image segmentation method, VMPANet. We integrated Progressive Contextual Extraction (PCM), utilizing pixel correlations to gradually extract global and local contextual information and achieve in-depth exploration of salient regions based on visual Mamba. Additionally, we incorporated prompt generation features to create mask prompts that assist in feature propagation, enhancing the model’s ability to accurately identify and refine important targets. Furthermore, we introduced the Multi-scale Contextual Attention (MCA) module, which employs parallel depthwise separable convolutions for multi-scale feature extraction, effectively capturing contextual information and regions of interest from edge, spatial, and channel perspectives. This approach is particularly suitable for handling medical images with complex backgrounds, blurred edges, and intricate targets. We also presented the Cross Attention Fusion (CAF) module, combining single-head self-attention mechanisms with cross-layer feature aggregation techniques across spatial dimensions. This addresses computational redundancy issues and enhances model performance. Experimental results demonstrate that VMPANet outperforms existing mainstream methods, achieving improvements of 1.38% and 0.83% in mIoU and DSC on the ISIC2017 dataset, and 1.10% and 0.67% on the ISIC2018 dataset, with only 0.383M parameters and 1.159 GFLOPs. In future work, we aim to develop more efficient and lightweight models and explore advanced techniques further.

Despite these encouraging results, this study has some inherent limitations that warrant attention: the model adapts poorly to extreme cases (such as very small lesions or severely blurred boundaries) and has not yet been fully integrated into clinical workflows for practical application validation. To address these shortcomings, future work will focus on expanding multi-source clinical data to enhance the model’s generalization ability, optimizing feature modeling for extreme cases, and exploring integration with clinical workflows, thereby further improving the practical value of this approach in clinical practice.

## Figures and Tables

**Table 2:** Composition of ISIC2017 and ISIC2018 datasets.

Dataset	Total Number of Images	Training Set Size	Test Set Size
ISIC2017	2150	1500	650
ISIC2018	2694	1886	808
PH2	All 200 images were used for external validation		

**Table 3:** Depth supervision weight settings on ISIC17 and ISIC18. Bold text is used to highlight key performance indicators.

	Parameter Settings	ISIC2017				
		mIoU (%)	DSC (%)	Acc (%)	Spe (%)	Sen (%)
**1**	**[1,0.75,0.5,0.25,0.1]**	77.50	86.32	95.21	**97.65**	85.12
**2**	**[0.1,0.25,0.5,0.75,1]**	77.38	86.20	95.10	97.22	86.85
**3**	**[1,1,1,1,1]**	**79.85**	**88.42**	**95.98**	97.58	**88.90**
	**Parameter settings**	**ISIC2018**				
		**mIoU (%)**	**DSC (%)**	**Acc (%)**	**Spe (%)**	**Sen (%)**
**1**	**[1,0.75,0.5,0.25,0.1]**	77.85	86.72	93.65	96.73	85.74
**2**	**[0.1,0.25,0.5,0.75,1]**	77.62	86.55	93.52	96.41	86.28
**3**	**[1,1,1,1,1]**	**80.32**	**88.80**	**94.48**	**97.35**	**87.15**

**Table 4:** Performance of the sixteen methods on the ISIC17 dataset. Bold text is used to highlight key performance indicators.

Algorithm	Year	mIoU (%)	DSC (%)	Acc (%)	Spe (%)	Sen (%)	HD95
**UNet [2]**	2015	73.38 ± 0.25	84.55 ± 0.22	95.01 ± 0.12	97.68 ± 0.10	81.75 ± 0.20	15.82 ± 1.67
**nnU-Net [41]**	2021	78.62 ± 0.18	87.85 ± 0.15	95.85 ± 0.09	97.58 ± 0.07	87.25 ± 0.13	12.85 ± 1.22
**SwinUnet [10]**	2022	73.72 ± 0.21	84.78 ± 0.18	95.08 ± 0.10	97.65 ± 0.09	82.05 ± 0.18	15.36 ± 1.55
**TransUnet [11]**	2021	77.52 ± 0.18	87.28 ± 0.15	95.78 ± 0.08	97.65 ± 0.17	86.38 ± 0.15	13.85 ± 1.32
**Hiformer [13]**	2023	79.02 ± 0.12	88.25 ± 0.10	96.05 ± 0.06	97.68 ± 0.05	88.05 ± 0.11	13.28 ± 1.25
**H-vmunet [16]**	2025	78.75 ± 0.15	88.02 ± 0.12	96.08 ± 0.07	**98.05 ± 0.06**	86.12 ± 0.13	12.68 ± 1.18
**VM-Unet [29]**	2024	79.34 ± 0.16	88.42 ± 0.11	96.15 ± 0.14	97.85 ± 0.15	87.65 ± 0.12	12.52 ± 1.15
**SkinMamba [31]**	2024	78.95 ± 0.14	88.15 ± 0.10	96.05 ± 0.10	97.72 ± 0.11	87.68 ± 0.14	12.40 ± 1.12
**EMCAD [23]**	2024	77.65 ± 0.17	87.38 ± 0.14	95.82 ± 0.09	97.68 ± 0.08	86.42 ± 0.16	13.56 ± 1.28
**MHorUNet [24]**	2024	78.15 ± 0.16	87.68 ± 0.13	95.98 ± 0.07	98.00 ± 0.16	85.72 ± 0.15	12.88 ± 1.15
**Ultralight vm-unet [42]**	2024	77.59 ± 0.18	87.95 ± 0.17	95.89 ± 0.13	97.74 ± 0.16	86.56 ± 0.18	13.41 ± 1.35
**U-Mamba [14]**	2024	79.27 ± 0.16	88.30 ± 0.15	95.74 ± 0.12	97.71 ± 0.15	88.10 ± 0.16	12.67 ± 1.20
**DermoMamba [43]**	2025	79.33 ± 0.14	88.39 ± 0.13	95.88 ± 0.11	97.80 ± 0.14	88.07 ± 0.15	12.38 ± 1.05
**UD-Mamba [44]**	2025	79.51 ± 0.13	88.50 ± 0.12	96.04 ± 0.10	97.91 ± 0.13	88.12 ± 0.14	12.13 ± 1.02
**MCM-UNet [45]**	2026	78.92 ± 0.15	88.21 ± 0.14	95.94 ± 0.12	91.61 ± 0.18	87.96 ± 0.16	12.56 ± 1.20
**VMPANet (Our)**		**80.72 ± 0.08**	**89.25 ± 0.06**	**96.38 ± 0.11**	97.68 ± 0.10	**89.85 ± 0.07**	**12.05 ± 0.85**

**Table 5:** Performance of sixteen methods on ISIC18 dataset. Bold text is used to highlight key performance indicators.

Algorithm	Year	mIoU (%)	DSC (%)	Acc (%)	Spe (%)	Sen (%)	HD95
**UNet [2]**	2015	77.12 ± 0.23	86.95 ± 0.20	93.58 ± 0.11	95.35 ± 0.09	88.02 ± 0.18	16.15 ± 1.72
**nnU-Net [41]**	2021	78.95 ± 0.16	88.12 ± 0.13	93.85 ± 0.08	95.95 ± 0.11	86.05 ± 0.12	13.18 ± 1.25
**SwinUnet [10]**	2022	74.82 ± 0.20	85.52 ± 0.17	92.95 ± 0.10	95.65 ± 0.08	84.78 ± 0.17	15.68 ± 1.60
**TransUnet [11]**	2021	78.15 ± 0.17	87.68 ± 0.14	93.98 ± 0.09	96.15 ± 0.08	87.28 ± 0.14	14.12 ± 1.35
**Hiformer [13]**	2023	79.92 ± 0.11	88.78 ± 0.09	94.43 ± 0.06	95.85 ± 0.07	87.92 ± 0.10	13.55 ± 1.29
**H-vmunet [16]**	2025	79.98 ± 0.12	88.82 ± 0.10	94.62 ± 0.08	97.15 ± 0.06	86.82 ± 0.11	12.88 ± 1.17
**VM-Unet [29]**	2024	79.95 ± 0.13	88.82 ± 0.10	94.44 ± 0.06	96.42 ± 0.15	88.65 ± 0.11	12.75 ± 1.20
**SkinMamba [31]**	2024	78.72 ± 0.15	88.02 ± 0.12	94.25 ± 0.07	96.95 ± 0.06	85.82 ± 0.13	12.62 ± 1.18
**EMCAD [23]**	2024	80.05 ± 0.12	88.85 ± 0.09	94.52 ± 0.09	95.72 ± 0.09	88.45 ± 0.10	13.82 ± 1.30
**MHorUNet [24]**	2024	79.62 ± 0.14	88.62 ± 0.11	94.32 ± 0.12	95.65 ± 0.08	**89.08 ± 0.12**	13.15 ± 1.20
**Ultralight vm-unet [42]**	2024	78.40 ± 0.17	87.65 ± 0.16	94.12 ± 0.13	95.77 ± 0.17	88.03 ± 0.17	13.43 ± 1.38
**U-Mamba [14]**	2024	79.87 ± 0.15	88.14 ± 0.14	94.36 ± 0.12	96.27 ± 0.15	88.22 ± 0.15	13.21 ± 1.25
**DermoMamba [43]**	2025	79.89 ± 0.13	88.71 ± 0.12	94.49 ± 0.11	96.51 ± 0.14	88.30 ± 0.14	12.88 ± 1.08
**UD-Mamba [44]**	2025	80.10 ± 0.12	88.87 ± 0.11	94.57 ± 0.10	96.91 ± 0.13	88.45 ± 0.13	12.31 ± 1.05
**MCM-UNet [45]**	2026	79.11 ± 0.16	87.79 ± 0.15	94.26 ± 0.13	95.99 ± 0.17	88.07 ± 0.16	13.30 ± 1.28
**VMPANet (Our)**		**81.15 ± 0.06**	**89.52 ± 0.07**	**95.02 ± 0.10**	**97.52 ± 0.06**	87.25 ± 0.08	**12.28 ± 0.95**

**Table 6:** The PH2 dataset was compared with the external validation results of mainstream models. The PH2 dataset is a zero-shot external validation, performed only once, with no standard deviation. Bold text is used to highlight key performance indicators.

Algorithm	Year	mIoU (%)	DSC (%)	Acc (%)	Spe (%)	Sen (%)	HD95
**UNet [2]**	2015	81.44	88.35	92.10	92.31	94.27	15.58
**nnU-Net [41]**	2021	83.40	90.55	93.28	93.07	95.38	13.47
**SwinUnet [10]**	2022	82.93	89.95	93.07	93.02	95.24	14.15
**TransUnet [11]**	2021	83.28	90.27	93.32	93.24	95.43	13.78
**Hiformer [13]**	2023	83.65	90.64	93.61	93.38	95.57	13.32
**H-vmunet [16]**	2025	84.89	91.88	94.45	93.82	96.05	11.59
**VM-Unet [29]**	2024	84.62	91.65	94.31	93.74	95.97	11.87
**SkinMamba [31]**	2024	84.37	91.38	94.17	93.62	95.85	12.25
**EMCAD [23]**	2024	84.02	91.03	93.94	93.46	95.69	12.79
**MHorUNet [24]**	2024	84.15	91.16	93.99	93.51	95.73	12.64
**Ultralight vm-unet [42]**	2024	84.23	91.24	94.05	93.55	95.78	12.48
**U-Mamba [14]**	2024	84.49	91.51	94.23	93.68	95.91	12.03
**DermoMamba [43]**	2025	85.13	92.03	94.56	93.88	96.12	11.32
**UD-Mamba [44]**	2025	85.42	92.18	**94.79**	93.96	96.19	11.05
**MCM-UNet [45]**	2026	85.27	92.11	94.63	93.92	96.15	11.21
**VMPANet (Our)**		**85.60**	**92.24**	94.78	**94.08**	**96.24**	**10.57**

**Table 7:** Model size comparison (GFLOPs/M). Bold text is used to highlight key performance indicators.

Algorithm	Computational Cost	Parameters
UNet [2]	40.111	17.263
nnU-Net [41]	45.607	19.312
SwinUnet [10]	8.693	41.380
TransUnet [11]	24.670	105.276
Hiformer [13]	7.522	29.518
H-vmunet [16]	0.742	8.973
VM-Unet [29]	4.112	27.428
SkinMamba [31]	5.093	14.083
EMCAD [23]	5.596	26.764
MHorUNet [24]	0.575	4.962
Ultralight vm-unet [42]	**0.060**	**0.049**
U-Mamba [14]	4.232	28.124
DermoMamba [43]	0.970	4.950
UD-Mamba [44]	5.910	19.120
MCM-UNet [45]	0.140	0.600
VMPANet (Our)	1.159	0.383

**Table 8:** Shows the parameters and computational cost of the model’s encoder, decoder, and PH (segmentation head).

Module	Parameter Count (M)	FLOPs (G)	Proportion
Encoder	0.135	0.328	28.5%
Decoder (including CAF)	0.242	0.816	70.4%
PH (segmentation head)	0.006	0.015	1.1%
Total	0.383	1.159	100%

**Table 9:** Ablation studies on ISIC17 and ISIC18 datasets.

	PCM	MCA	CAF	ISIC2017				
				mIoU (%)	DSC (%)	Acc (%)	Spe (%)	Sen (%)
**1**	✓			79.04 ± 0.14	88.15 ± 0.08	95.99 ± 0.10	97.50 ± 0.09	88.46 ± 0.08
**2**	✓	✓		79.48 ± 0.12	88.59 ± 0.12	96.09 ± 0.09	97.69 ± 0.11	88.59 ± 0.07
**3**	✓	✓	✓	80.72 ± 0.05	89.25 ± 0.10	96.38 ± 0.08	97.68 ± 0.07	89.85 ± 0.06
	**PCM**	**MCA**	**CAF**	**ISIC2018**				
				**mIoU (%)**	**DSC (%)**	**Acc (%)**	**Spe (%)**	**Sen (%)**
**1**	✓			79.47 ± 0.17	88.47 ± 0.11	94.08 ± 0.11	96.44 ± 0.12	86.93 ± 0.10
**2**	✓	✓		80.06 ± 0.11	88.95 ± 0.09	94.33 ± 0.07	96.73 ± 0.07	87.35 ± 0.07
**3**	✓	✓	✓	81.15 ± 0.06	89.52 ± 0.07	95.02 ± 0.10	97.52 ± 0.09	87.25 ± 0.08

**Table 10:** Component-Level Ablation Results (ISIC2017 & ISIC2018).

Model Configuration	ISIC2017			ISIC2018		
	mIoU (%)	DSC (%)	HD95	mIoU (%)	DSC (%)	HD95
1. Full VMPANet	80.72 ± 0.08	89.25 ± 0.06	12.05 ± 0.85	81.15 ± 0.06	89.52 ± 0.07	12.28 ± 0.95
2. PCM w/o Mamba	79.87 ± 0.08	88.40 ± 0.12	12.68 ± 0.92	80.22 ± 0.14	88.65 ± 0.11	12.95 ± 1.02
3. PCM w/o Prompt	80.15 ± 0.07	88.75 ± 0.11	12.42 ± 0.88	80.58 ± 0.08	88.98 ± 0.10	12.63 ± 0.98
4. MCA w/o EE	80.28 ± 0.10	88.92 ± 0.10	12.77 ± 0.90	80.72 ± 0.17	89.15 ± 0.09	13.00 ± 1.00
5. MCA w/o SA	79.95 ± 0.09	88.58 ± 0.13	12.95 ± 0.95	80.35 ± 0.11	88.82 ± 0.12	13.18 ± 1.05
6. MCA w/o CA	80.02 ± 0.11	88.65 ± 0.12	12.88 ± 0.93	80.42 ± 0.09	88.90 ± 0.11	13.12 ± 1.03
7. w/o CAF	79.65 ± 0.14	88.32 ± 0.14	13.25 ± 0.98	80.05 ± 0.13	88.55 ± 0.13	13.45 ± 1.08

## Data Availability

The data presented in this study are openly available in [ISIC] at [https://challenge.isic-archive.com/data/] (accessed on 15 November 2024).
